# Comparison of hybrid surgeries for the treatment of three-level cervical degenerative disease: a systematic review and network meta-analysis

**DOI:** 10.3389/fsurg.2026.1882426

**Published:** 2026-07-08

**Authors:** Jiyuan Liao, Yuchen Duan, Yunfei Lu, Wenjie Liu

**Affiliations:** 1 Department of Neurosurgery, West China Hospital, Sichuan University/West China School of Nursing, Sichuan University, Chengdu, China; 2 West China School of Nursing, Sichuan University, Chengdu, China; 3 Department of Orthopedics, Hospital of Chengdu University of Traditional Chinese Medicine, Chengdu, Sichuan Province, China

**Keywords:** anterior cervical discectomy and fusion, cervical disc replacement, hybrid surgery, network meta-analysis, three-level cervical degenerative disease

## Abstract

**Background:**

In recent years, hybrid surgery (HS) has emerged as one of the major surgical modalities for the treatment of multisegmental cervical degenerative disease (CDD). Two types of HS have been introduced (HS1, 1-level TDR with 2-level anterior cervical discectomy and fusion, ACDF or HS2, 2-level TDR with 1-level ACDF), and the clinical and radiological results could differ.

**Methods:**

This study was conducted according to the Preferred Reporting Items for Systematic Reviews and Meta-Analyses (PRISMA) Statement. The Web of Science Core Collection, PubMed and Embase were comprehensively searched from inception to December 19, 2025.

**Results:**

Five studies were included in this network meta-analysis. The Neck disability index (NDI) in the HS1 was significantly lower than that in the ACDF. The C2-7 range of motions (ROMs) in the HS1 and HS2 were both significantly greater than those in the ACDF. The C2-7 ROM in the HS1 was significantly lower than that in the HS2. The ROMs of the upper and lower segments in the HS1 and HS2 were both significantly lower than those in the ACDF. No significant difference was identified in the ROM of the upper or lower segment between the HS1 and the HS2.

**Conclusion:**

HS1, HS2 and ACDF are all safe and effective surgical options for three-level CDD. HS1 and HS2 could better preserve the C2-7 ROM. ACDF is associated with an increased ROM of adjacent segments. Compared with HS1, HS2 could better preserve the C2-7 ROM. HS1, HS2 and ACDF presented similar incidences of ASD.

**Systematic Review Registration:**

https://www.crd.york.ac.uk/PROSPERO/view/CRD420251271417, PROSPERO (CRD420251271417).

## 1 Introduction

Cervical degenerative disease (CDD) refers to a category of disorders triggered by degenerative pathological changes in the cervical spine. Its pathogenesis is mainly attributed to chronic cervical strain, osteophyte formation, intervertebral disc herniation, ligament thickening, and other factors, which consequently compress the cervical spinal cord, nerve roots, or vertebral artery (1). Anterior cervical discectomy and fusion (ACDF), which pioneered a new era in the surgical management of CDD, was first proposed by Smith and Robinson in 1958 (2). Despite the passage of more than sixty years, it remains the gold standard for the surgical treatment of this condition.

The ACDF has also been widely adopted for the management of multisegmental CDD (3). Swank et al. (4) reported that the pseudoarthrosis rate was 36% for 2-level ACDF and as high as 54% for 3-level ACDF. Lowery et al. (5) reported that the incidence of implant-related complications such as plate breakage or screw loosening was 20% after single-level fusion and 36% after 2-level fusion, whereas the rates increased to 71% and 80% after 3-level and 4-level fusion, respectively. Moreover, long-segment fusion has been identified as a crucial risk factor for adjacent segment degenetation (ASD) in relevant studies. Specifically, the incidence of ASD was 13.2% following single-level ACDF vs. 32.1% following multilevel ACDF (6). The development of ASD often leads to recurrent clinical symptoms and increases the risk of revision surgery (7). In addition, the literature has confirmed that the revision surgery rate increases with increasing number of fused segments: the rate was 5.8% after single-level ACDF, 19% after 2-level ACDF (8), and as high as 35% after 3-level or 4-level ACDF (7).

Although anterior cervical fusion can restore cervical spine stability, previous studies have reported that long-segment fusion is associated with an increased incidence of complications. In multisegment CDD, the degree of degeneration varies among different segments. Surgeons can select surgical approaches in a targeted manner according to the severity of degeneration of the affected segments: total disc replacement (TDR) is performed on segments dominated by soft disc herniation with preserved mobility, whereas ACDF is adopted for segments with osteophyte formation and severe degeneration (9). In recent years, hybrid surgery (HS) has emerged as one of the major surgical modalities for the treatment of multisegmental CDD (10).

Yang et al. (11) compared the safety and efficacy of ACDF with those of HS for two-level CDD and demonstrated that HS was associated with better radiological results and similar clinical results. Ragab et al. (10) also conducted a meta-analysis to compare ACDF with HS for multilevel CDD. They reported that HS was associated with better C2-7 range of motion (ROM), a lower ROM of adjacent segments and a similar incidence of ASD. However, both two-level and three-level CDD were included, and several studies included in this review were missed. No meta-analysis comparing HS and ACDF for the treatment of 3-level CDD has been published to date. Moreover, two types of HS have been reported in previous studies (HS1, 1-level TDR with 2-level ACDF or HS2, 2-level TDR with 1-level ACDF), and the clinical and radiological results could differ. HS group in Ragab et al. (10) was not subdivided into HS1 group and HS2 group. Thus, we performed this systematic review and network meta-analysis to compare the safety and efficacy of HS1, HS2 and ACDF for three-level CDD.

## 2 Materials and methods

### 2.1 Study registration and search strategy

This study was prospectively registered at PROSPERO (CRD420251271417). This systematic review and network meta-analysis was conducted according to the PRISMA Statement (12, 13). No amendment was done to information provided at registration. The Web of Science Core Collection, PubMed and Embase databases were comprehensively searched from inception to December 19, 2025, for relevant randomized controlled trials (RCTs) and prospective and retrospective cohort studies. These terms, including (three level* OR three-level OR 3-level OR 3 level* OR multi-level OR multilevel*) AND (total dis* replacement OR cervical dis* arthroplasty OR artificial cervical dis* replacement OR TDR OR CDA OR ACDR OR artificial dis* replacement OR hybrid surgery OR hybrid cervical surgery) AND (anterior cervical dis*ectomy and fusion OR ACDF), were applied in the literature search in the title or abstract. We applied no language restrictions.

### 2.2 Inclusion and exclusion criteria

Articles that met the following criteria were included: (1) patients clinically confirmed with three-level CDD, which was confirmed by CT or MRI; (2) failure to achieve clinical improvement following no less than 6 weeks of conservative therapy and surgical intervention via the anterior cervical interspace approach; (3) studies comparing the safety, clinical and radiological outcomes among at least 2 of the 3 surgical options (ACDF, HS1 or HS2) for the treatment of three-level CDD; and (3) RCTs, quasi-RCTs, cohort studies. Articles that met these criteria were excluded: (1) noncomparative studies; (2) anterior corpectomy was applied;(3) case reports; (4) reviews; (5) conference abstracts; (6) biomechanical studies; (7) finite element studies; and (8) studies that included 2- to 4-level CDD patients, and the data of 3-level CDD patients could not be separated.

### 2.3 Data extraction

The following information was collected: (1) basic data such as publication year, first author, country, age, sex and follow-up time; (2) clinical data such as VAS score, NDI score, JOA score and complication rate; (3) radiological data such as C2‒7 Cobb angle, C2-7 ROM, upper and lower segment ROM, ASD and fusion rate; and (5) methodological data extracted by two authors and then verified by a third author.

### 2.4 Risk of bias assessment

The NOS was employed to assess the methodological quality of cohort studies (14) covering the following three dimensions: selection of patients, comparability among the different groups and evaluation of outcomes. The total score of the NOS was 9.

### 2.5 Statistical analysis

The network meta-analysis was performed via STATA18.0 (StataCorp, Texas). The C2-7 Cobb angle, C2-7 ROM, ROM of the upper and lower segments, VAS score for arm and neck, NDI score and JOA score were analyzed via the mean difference (MD) and 95% confidence interval (CI). Dichotomous data, including the complication rate, incidence of ASD and fusion rate, were analyzed for relative risk (RR) and 95% CI. *P* < 0.05 was considered significant. Sensitivity analysis was conducted to evaluate the reliability of the outcome. The funnel plot was adopted to assess the small-study effect. The global, local and loop inconsistency were analyzed. The ‘network map’ procedure was applied to present the network relationships among the included studies. The ’sucra’ procedure was conducted to rank the three surgical options (three-level ACDF or one-level TDR with two-level ACDF or two-level TDR with one-level ACDF) through the value of the surface under the cumulative ranking curve (SUCRA).

## 3 Results

### 3.1 Identification of relevant studies

**Figure 1:**
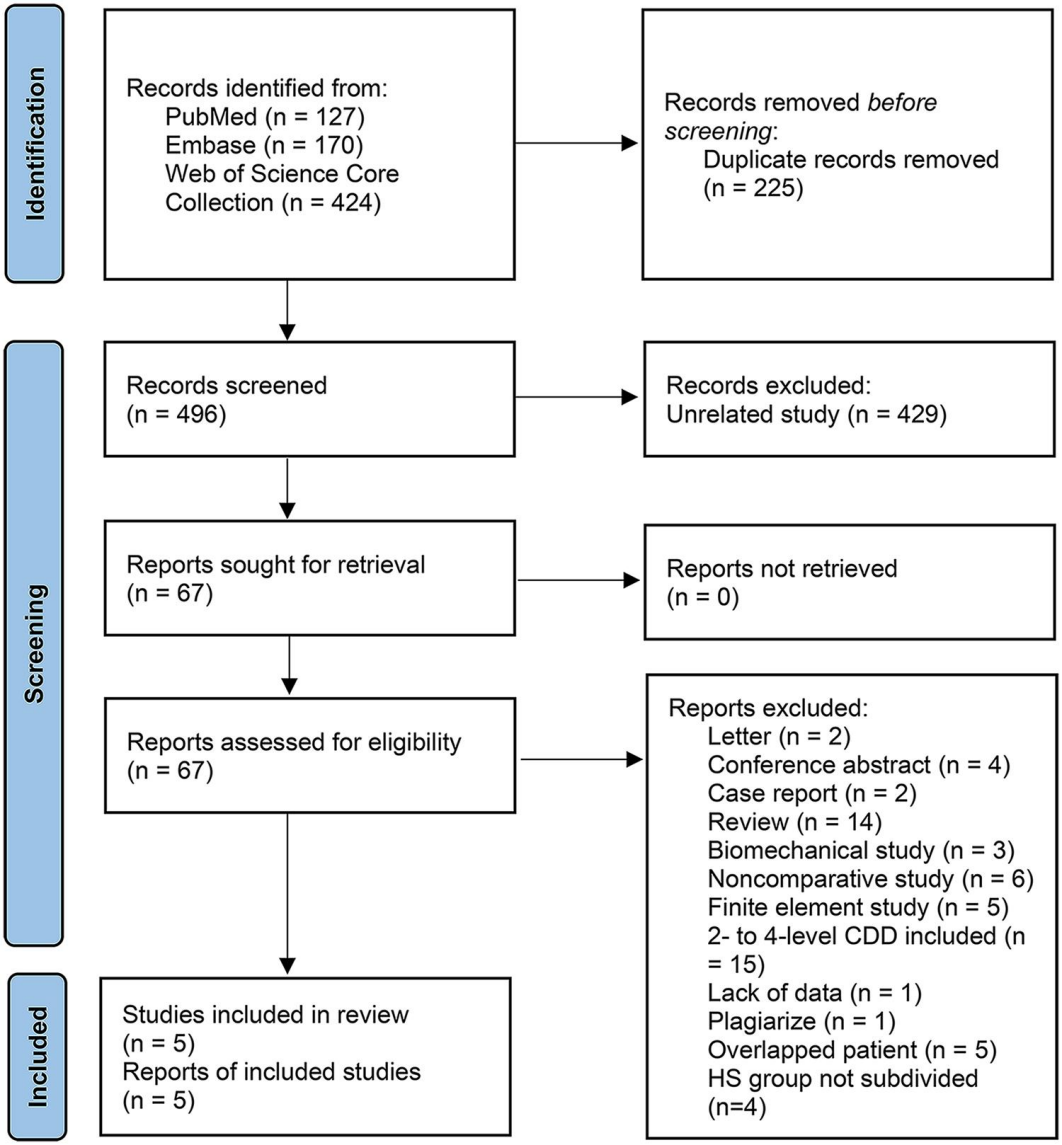
A flow diagram of study selection.

We identified 721 studies through PubMed, the Web of Science Core Collection and Embase. Two hundred and twenty-five duplicates were removed, and 496 studies were ultimately retrieved. There were 429 unrelated studies, 2 letters, 4 conference abstracts, 6 noncomparative studies, 5 finite element studies, 14 reviews, 2 case reports and 3 biomechanical studies excluded. Fifteen studies were excluded because 2- to 4-level CDD were included, and 3-level CDD could not be separated. Four studies (15–18) were excluded because of overlapping patients. One study (19) was excluded for reporting only *p* values without providing the actual data. One study (20) was excluded for being identified as plagiarized because of the complete overlap of its data with those of another study (21). Four studies were excluded because the HS group was not subdivided into HS1 and HS2 (22–25). The two studies by Chen et al. (26) and Huang et al. (27) shared overlapping participants. Given the richer outcome data in Huang's research, Chen et al. (26) was excluded. Eventually, five studies (27–30) were included in this meta-analysis. There are two types of hybrid surgeries: one-level TDR with two-level ACDF (HS1 group) and two-level TDR with one-level ACDF (HS2 group). The study selection process is shown in Figure 1.

### 3.2 Characteristics of the included studies and quality assessment

**Figure 2:**
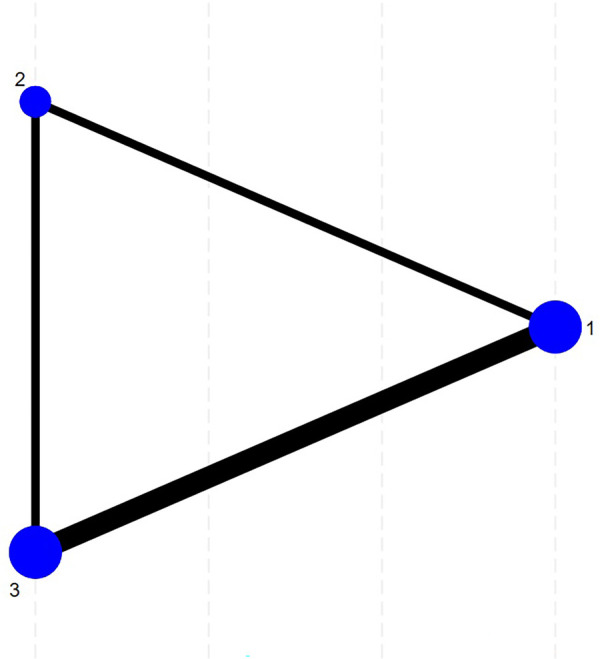
Network relationships among the comparisons for the network meta-analysis. 1: ACDF; 2: HS1; 3: HS2.

Five studies with a total of 308 patients who received one of three surgical options (ACDF, HS1, or HS2) for treating three-level CDD were identified. ACDF surgery was administered to 105 patients, whereas HS1 and HS2 surgical procedures were performed in 86 and 117 cases, respectively. The basic information is showed in Table 1. All included studies were assessed according to the NOS, the results of which are shown in Table 2. The mean score (ranging from 0 to 9) of all included studies was 7.8. The network relationships are shown in Figure 2**.**

**Table 1:** Basic information of the included studies.

Study	Year	Study design	Country	Treatment	Sample size	Mean age	Gender(M:F)	Follow-up(month)
Kang (21)	2013	Quasi-RCT	China	ACDF	12	55.3 ± 6.7	7:5	33.2 ± 7.7
				HS2	12	53.6 ± 6.1	8:4	32.8 ± 7.5
Chang (28)	2017	Retrospective	China	ACDF	17	44.35 ± 4.40	12:5	28.44 ± 19.2
				HS2	20	44.76 ± 5.73	17:3	28.44 ± 19.2
Xu (29)	2020	Retrospective	China	ACDF	32	57.2 ± 8.3	17:15	89.3 ± 9.5
				HS1	36	55.5 ± 7.5	17:19	93.3 ± 8.5
				HS2	25	55.2 ± 12.6	14:11	76.0 ± 6.3
Huang (27)	2022	Retrospective	China	ACDF	24	56.67 ± 7.93	13:11	29.13 ± 14.19
				HS1	50	51.83 ± 6.86	26:24	29.13 ± 14.40
				HS2	34	48.94 ± 8.93	17:17	34.03 ± 19.74
Qiu (30)	2025	Retrospective	China	ACDF	20	56.00 ± 8.54	8:12	142.4 ± 13.17
				HS2	26	51.81 ± 7.05	12:14	141.42 ± 20.00

**Table 2:** Methodological quality assessment of included studies according to NOS.

Domain	References	Kang (21)	Chang (28)	Xu (29)	Huang (27)	Qiu (30)
Selection	Representativeness of the exposed cohort	1	1	1	1	1
	Selection of the non-exposed cohort	1	1	1	1	1
	Ascertainment of exposure	1	1	1	1	1
	Demonstration that outcome of interest was not present at the start of study	1	1	1	1	1
Comparability	Study controls for level	1	1	1	1	1
	Study controls for any additional factor	1	1	1	1	1
Outcome	Assessment of outcome	1	1	1	1	1
	Follow-up long enough for outcomes to occur	0	0	1	0	1
	Adequacy of follow-up of cohort	0	0	1	0	1
Total		7	7	9	7	9

### 3.3 Network meta-analysis of outcomes

#### 3.3.1 Clinical outcomes

**Figure 3:**
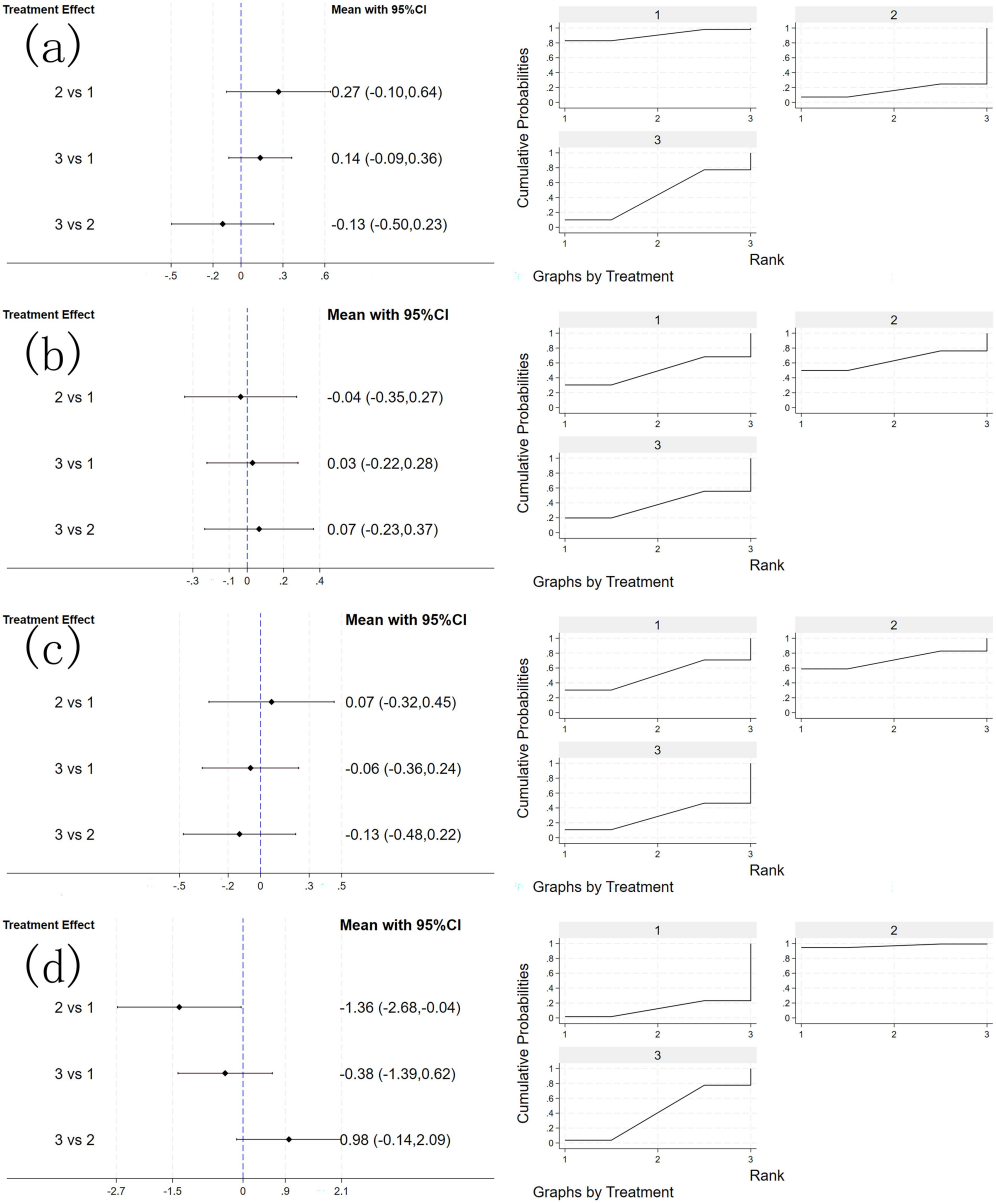
Comparison between ACDF and HS in terms of VAS score for arm **(a)**, VAS score for neck **(b)**, JOA score **(c)** and NDI score **(d)** 1 = A = ACDF; 2 = B = HS1; 3 = C = HS2.

The mean VAS scores for arm in the ACDF, HS1 and HS2 were 0.98, 0.87 and 0.93, respectively. No significant difference was found in the VAS score for arm between the ACDF and the HS1 (MD, 0.27; 95% CI, −0.10, 0.64), the ACDF and the HS2 (MD, 0.14; 95% CI, −0.09, 0.36), or the HS1 and the HS2 (MD, −0.13; 95% CI, −0.50, 0.23, Figure 3a). The SUCRA probabilities of ACDF (89.8%), HS1 (44.3%) and HS2 (15.9%) are presented in Figure 3a. The mean VAS scores for neck in the ACDF, HS1 and HS2 groups were 1.43, 0.87 and 1.21, respectively. No significant difference was found in the VAS score for neck between the ACDF and the HS1 (MD, −0.04; 95% CI, −0.35, 0.27), between the ACDF and the HS2 (MD, 0.03; 95% CI, −0.22, 0.28), or between the HS1 and the HS2 (MD, −0.07; 95% CI, −0.23, 0.37, Figure 3b). The SUCRA probabilities of ACDF (50.6%), HS1 (62.6%) and HS2 (36.8%) are presented in Figure 3b.

The mean JOA scores in the ACDF, HS1 and HS2 were 15.88, 16.2 and 15.9, respectively. No significant difference was found in the JOA score between the ACDF and the HS1 (MD, 0.07; 95% CI, −0.32, 0.45), between the ACDF and the HS2 (MD, −0.06; 95% CI, −0.36, 0.24), or between the HS1 and the HS2 (MD, −0.13; 95% CI, −0.48, 0.22, Figure 3c). The SUCRA probabilities of ACDF (51.2%), HS1 (69.7%) and HS2 (29.1%) are presented in Figure 3c.

The mean NDI scores in the ACDF, HS1 and HS2 were 9.6, 7.39 and 8.42, respectively. The NDI score in the HS1 was significantly lower than that in the ACDF (MD, −1.36; 95% CI, −2.68, −0.04). No significant difference was found in the NDI score between the ACDF and the HS2 (MD, −0.38; 95% CI, −1.39, 0.62) or between the HS1 and the HS2 (MD, 0.98; 95% CI, −0.14, 2.09, Figure 3d). The SUCRA probabilities of ACDF (12.1%), HS1 (97.0%) and HS2 (40.8%) are presented in Figure 3d.

#### 3.3.2 Complication rate

**Figure 4:**
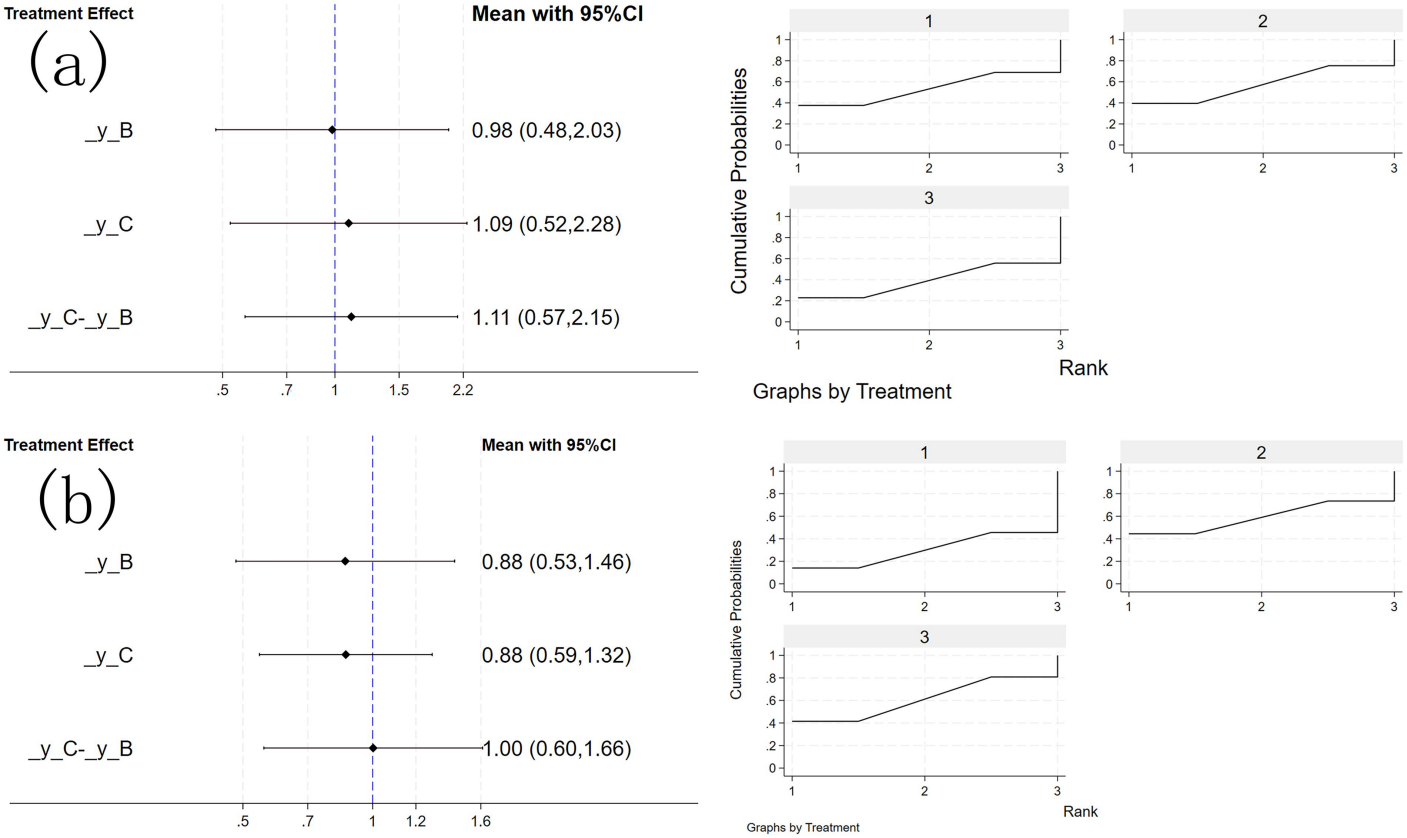
Comparison between ACDF and HS in terms of complication rate **(a)** and ASD **(b)** 1 = A = ACDF; 2 = B = HS1; 3 = C = HS2.

Of the five eligible studies, three presented data on complication rates (Chang et al. (28), Xu et al. (29) and Huang et al. 27). Thus, a total of 86 patients in HS1, 79 patients in HS2 and 73 patients in ACDF were included in the analysis in complication rate. The mean complication rates in the ACDF, HS1 and HS2 groups were 12.3%, 17.4% and 16.5%, respectively. No significant differences were found in the complication rates between the ACDF and the HS1 (RR, 0.98; 95% CI, 0.48, 2.03), the ACDF and the HS2 (RR, 1.09; 95% CI, 0.52, 2.28), or the HS1 and the HS2 (RR, 1.11; 95% CI, 0.57, 2.15, Figure 4a). The SUCRA probabilities of ACDF (53.4%), HS1 (57.4%) and HS2 (39.3%) are presented in Figure 4a.

#### 3.3.3 ASD

The mean incidence rates of ASD in the ACDF, HS1 and HS2 groups were 44.4%, 61.1% and 37.3%, respectively. No significant difference was found in the incidence of ASD between the ACDF and the HS1 (RR, 0.88; 95% CI, 0.53, 1.46), the ACDF group and the HS2 group (RR, 0.88; 95% CI, 0.59, 1.32), or the HS1 group and the HS2 (RR, 1.00; 95% CI, 0.60, 1.66, Figure 4b). The SUCRA probabilities of ACDF (29.6%), HS1 (58.6%) and HS2 (61.9%) are presented in Figure 4b.

#### 3.3.4 Radiological outcomes

**Figure 5:**
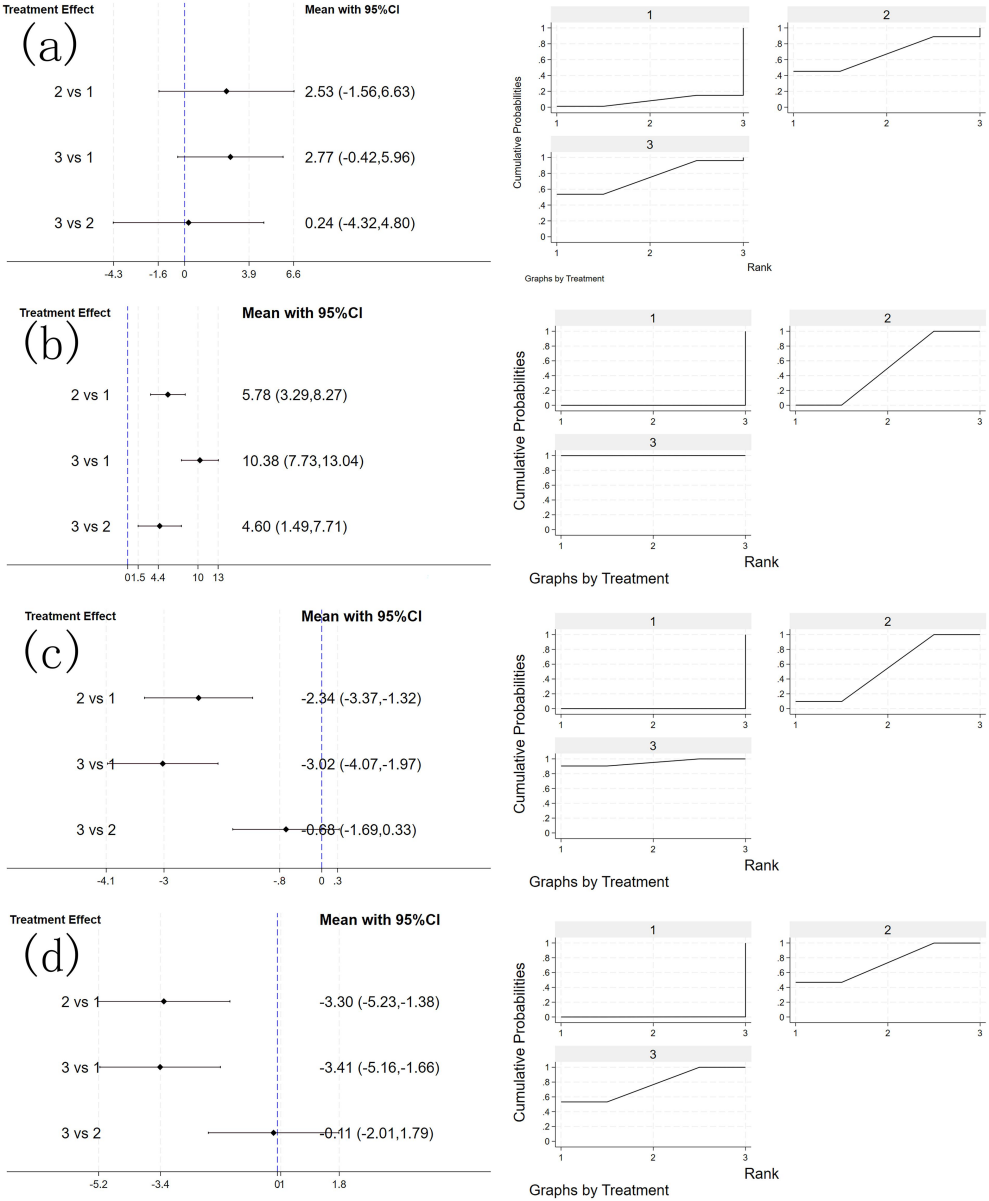
Comparison between ACDF and HS in terms of C2-7 cobb angle **(a)**, C2-7 ROM **(b)**, ROM of the upper segment **(c)** and ROM of the lower segment **(d)** 1 = A = ACDF; 2 = B = HS1; 3 = C = HS2.

The mean C2-7 Cobb angles in the ACDF, HS1 and HS2 groups were 11.78, 16.30 and 14.59, respectively. No significant difference was found in the C2-7 Cobb angle between the ACDF and HS1 (MD, 2.53; 95% CI, −1.56, 6.63), ACDF and HS2 (MD, 2.77; 95% CI, −0.42, 5.96), HS1 and HS2 (MD, 0.24; 95% CI, −4.32, 4.80, Figure 5a). The SUCRA probabilities of ACDF (7.7%), HS1 (66.9%) and HS2 (75.4%) are presented in Figure 5a.

The mean C2-7 ROM in the ACDF, HS1 and HS2 groups were 24.81, 27.73 and 34.87, respectively. The C2-7 ROM in the HS1 (MD, 5.78; 95% CI, 3.29, 8.27) and HS2 (MD, 10.38; 95% CI, 7.73, 13.04) were both significantly greater than those in the ACDF. The C2-7 ROM in the HS1 was significantly lower than that in the HS2 (MD, 4.60; 95% CI, 1.49, 7.71, Figure 5b). The SUCRA probabilities of ACDF (0.0%), HS1 (50.1%) and HS2 (99.9%) are presented in Figure 5b.

The mean ROMs of the upper segment in the ACDF, HS1 and HS2 groups were 12.73, 9.08 and 9.68, respectively. The ROMs of the upper segment in the HS1 (MD, −2.34; 95% CI, −3.37, −1.32) and HS2 (MD, −3.02; 95% CI, −4.07, −1.97) were both significantly lower than those in the ACDF. No significant difference was identified in the ROM of the upper segment between the HS1 and the HS2 (MD, −0.60; 95% CI, −1.09, 0.33, Figure 5c). The SUCRA probabilities of ACDF (0.0%), HS1 (54.8%) and HS2 (95.2%) are presented in Figure 5c. The mean ROMs of the lower segment in the ACDF, HS1 and HS2 groups were 11.38, 7.41 and 8.15, respectively. The ROMs of the lower segment in the HS1 (MD, −3.30; 95% CI, −5.23, −1.38) and HS2 (MD, −3.41; 95% CI, −5.16, −1.66) were both significantly lower than those in the ACDF. No significant difference was identified in the ROM of the lower segment between the HS1 and the HS2 (MD, −0.11; 95% CI, −2.01, 1.79, Figure 5d). The SUCRA probabilities of ACDF (0.0%), HS1 (73.3%) and HS2 (96.7%) are presented in Figure 5d.

### 3.4 Publication bias assessment, inconsistency test and sensitivity analysis

**Figure 6:**
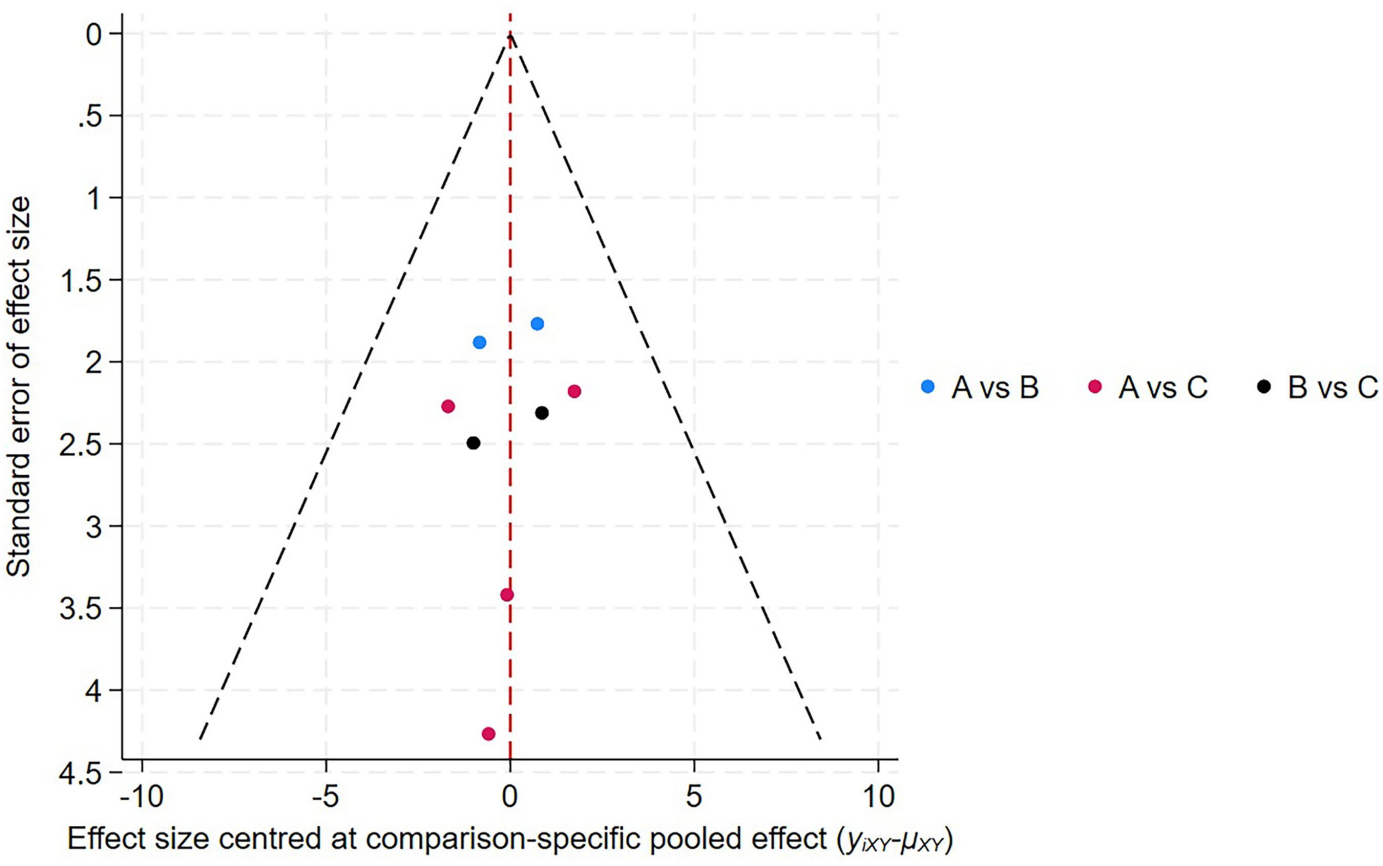
Funnel plot of this meta-analysis. 1 = A = ACDF; 2 = B = HS1; 3 = C = HS2.

No significant asymmetry was identified in the funnel plot, which demonstrated no small-study effect was identified (Figure 6). The 95% CIs of the loop crossed zero, which indicated that no significant loop inconsistency was identified. To gauge the stability of the overall results, a sensitivity analysis was carried out. When each study was excluded one by one from the analytical model, no substantial changes were detected, which confirmed that the conclusions were credible.

## 4 Discussion

This is the first network meta-analysis to compare HS1, HS2 and ACDF. Seven patients complained of dysphagia, 1 patient complained of hoarseness, 1 patient complained of reoperation due to compression in the ACDF group. Thirteen patients complained of dysphagia and 2 patients complained of cervical disc lock in the HS1 group. One patient complained of C5 palsy, one patient complained of hoarseness, 10 patients complained of dysphagia and one patient complained of cervical disc lock in the HS2 group. The complication rates were 17.4% (15/86), 16.5% (13/79) and 12.3% (9/73) in the HS1, HS2 and ACDF, respectively. No significant difference was detected. In summary, both HS surgery and ACDF surgery are safe procedures for three-level CDD.

The NDI score in the HS1 was significantly lower than ACDF. Deng et al. (9) enrolled 51 patients who underwent three-level HS between January 2018 and May 2020 and matched them 1:1 with patients who received posterior laminoplasty on the basis of factors such as age and sex for comparison. The results revealed that the clinical outcomes were significantly improved compared with those before surgery, with no statistical differences between the two groups. Visocchi et al. (31) retrospectively reviewed 62 patients who underwent three-level hybrid surgery and reported that the VAS, mJOA and Nurick scores in both the myelopathy and nonmyelopathy groups were significantly improved at the last follow-up. Zhang et al. (32) performed a meta-analysis to compare HS and ACDF for two- and three-level CDD and demonstrated that the NDI score in the HS was significantly lower than ACDF. The HS also presented a trend toward superiority over the ACDF in terms of the VAS score (*P* = 0.058). Gökoğlu et al. (33) also found that compared with one-level ACDF, three-level ACDF was associated with poor improvement of JOA score. This finding also highlights the need to investigate HS in managing multilevel CDD. These findings further corroborate the results of the present study. Both HS and ACDF were effective procedures for three-level CDD, and HS tended to outperform ACDF.

Compared with the ACDF, both the HS1 and the HS2 achieved better restoration of cervical lordosis. But the difference was not statistically significant. Chen et al. (26) reported that the C2-7 Cobb angle segmental Cobb angle of ACDF group was significantly lower than that in HS1 and HS2 group. However, Xu et al. (29) demonstrated that no statistical difference was identified in cervical lordosis among these three groups. In terms of the C2-7 ROM, the HS2 group exhibited optimal preservation of motion, followed by the HS1 group, whereas the ACDF group presented the greatest loss of cervical range of motion. Compared with the ACDF, both the HS1 and the HS2 had greater reductions in the ROMs of the adjacent segments. Liao et al. (34) divided 18 human cadaveric spines into three groups, and three-level ACDF, HS1 and HS2 were performed. They reported that the segmental ROM in the HS2 was not significantly different from that in the intact spine. The segmental ROM in the HS1 was significantly lower than HS2 but significantly greater than ACDF. Compared with the HS1 and the HS2, the ACDF significantly increased the ROM of the adjacent segment. Bhatt et al. (35) retrospectively analyzed 100 patients who performed two- or three-level HS and ACDF. They reported that the C2-7 ROM in the HS was significantly greater than ACDF (*P* < 0.001), and the degree of motion loss in the HS was significantly lower than ACDF (*P* < 0.001). Moreover, the ROM of the upper adjacent segment was notably lower than the preoperative baseline in the HS cohort, while the corresponding ROM in the ACDF cohort was markedly higher than its preoperative value, which indicated that the HS strategy could protect the adjacent segment. Qiu et al. (36) also compared the ACDF with the HS and reported that the HS was superior to the ACDF concerning the maintenance of C2-7 ROM. They also demonstrated that cervical lordosis could be related to axial symptoms after surgery. Findings from both biomechanical and clinical studies have demonstrated that hybrid surgery can preserve C2-7 ROM and reduce the ROM at adjacent segments, thereby improving patients’ quality of life while providing protection for adjacent segments. Huang et al. (37) further divided the HS1 group into three groups: TDR-ACDF-ACDF (a), ACDF-TDR-ACDF (b) and ACDF-ACDF-TDR (c). They demonstrated that the C2-7 ROM in the type c group was significantly lower than that in the type a and type b group. Different positions of the artificial cervical disc in hybrid surgery have varying impacts on overall cervical spine kinematics, which requires further investigation.

Wong et al. (38) performed a finite element study to compare the biomechanical properties of three-level TDR, ACDF and six different types of HS according to the position of the hybrid levels. They demonstrated that the intradiscal pressure and facet force of the adjacent segment in the ACDF were significantly greater than HS, which could eventually lead to the occurrence of ASD. However, our results revealed no significant difference in the incidence of ASD among the three groups. Chen et al. (26) reported that the incidence of ASD in ACDF, HS1 and HS2 group were 42.4%, 31.9% and 26.9%, respectively. And no statistical difference was identified among these three groups. Qiu et al. (30) conducted a 10-year follow-up comparing the ACDF group and HS2 group, and they reported that no significant difference was found between the two groups in the incidence of ASD. But interestingly, they demonstrated that the severity of ASD in ACDF group was statistically greater than HS2 group. There remains considerable controversy regarding how HS affects the incidence of ASD, and further research is required. There are several possible reasons for this. ASD may itself be a natural degenerative process, and there are numerous confounding factors involved in its development. The protective effect of artificial cervical discs on adjacent segments is attenuated in hybrid surgery. A meta-analysis conducted by Zhao et al. (39) aimed to assess the effectiveness of TDR vs. ACDF in the management of contiguous two-level CDD, with results showing that incidence of ASD in the TDR cohort was significantly less than ACDF cohort. Badhiwala et al. (40) also demonstrated that the reoperation rate of adjacent segment in the TDR was significantly lower than ACDF at the five-year follow-up. However, the reported results of ASD in clinical studies concerning HS are inconsistent with those of finite element analysis and two-level TDR groups. Ragab et al. (10) performed a meta-analysis to compare the efficacy of HS and ACDF for two- and three-level CDD and demonstrated that there no significant difference was identified concerning ASD. Moreover, C3-6 and C4-7 are the main types of three-level CDD, and for multilevel cervical surgery, the operative segments themselves already cover the levels that are prone to degeneration (29, 41). Heterotopic ossification (HO) may also contribute to the incidence of ASD. With increasing follow-up duration, the occurrence of HO impairs the functionality of artificial cervical discs, thereby increasing the incidence of ASD (42). The pathogenesis of ASD is complex and requires further investigation.

## 5 Strengths and weaknesses

There were several strengths in this meta-analysis: (1) a network meta-analysis was conducted to compare three different surgical options (HS1, HS2 and ACDF); (2) the SUCRA was conducted to identify the differences among these three surgical options. However, several limitations of this study should be mentioned: (1) five studies (308 patients in aggregate) were included in the network meta-analysis, notably, the overall sample size was relatively limited. (2) The follow-up time of some studies was insufficient. (3) All included studies except one quasi-RCT were cohort studies. Thus, more RCTs with long-term follow-up are needed to confirm the conclusions of this meta-analysis.

## 6 Conclusion

HS1, HS2 and ACDF are all safe and effective surgical options for the treatment of CDD. HS1 and HS2 could better preserve the C2-7 ROM. The ACDF is associated with an increased ROM of adjacent segments. Compared with HS1, HS2 could better preserve the C2-7 ROM. HS1, HS2 and ACDF presented similar incidences of ASD.

## Data Availability

The original contributions presented in the study are included in the article/Supplementary Material, further inquiries can be directed to the corresponding author.
